# Oral antigen exposure in newborn piglets circumvents induction of oral tolerance in response to intraperitoneal vaccination in later life

**DOI:** 10.1186/s12917-015-0350-8

**Published:** 2015-03-07

**Authors:** J Alex Pasternak, Siew Hon Ng, Rachelle M Buchanan, Sonja Mertins, George K Mutwiri, Volker Gerdts, Heather L Wilson

**Affiliations:** Vaccine and Infectious Disease Organization (VIDO), University of Saskatchewan, 120 Veterinary Road, Saskatoon, SK S7N 5E3 Canada; Current address: Klinikum der Universität zu Köln, Institut für Medizinische Mikrobiologie, Immunologie und Hygiene, Goldenfelsstraße 19-21, 50935 Köln, Germany

**Keywords:** Piglets, Neonate, Ovalbumin, Oral, Tolerance

## Abstract

**Background:**

We previously determined that newborn piglets orally gavaged with Ovalbumin (OVA) responded to systemic OVA re-exposure with tolerance; if adjuvants were included in oral vaccine, piglets responded with antibody-mediated immunity (Vet Immunol Immunopathol 161(3–4):211–21, 2014). Here, we will investigate whether newborn piglets gavaged with a vaccine comprised of OVA plus unmethylated CpG oligodeoxynucleotides (CpG; soluble component; OVA/CpG) combined with OVA plus CpG encapsulated within polyphosphazene microparticles (MP; particulate component) responded with systemic and mucosal immunity. To monitor the response to systemic antigen re-exposure, piglets were i.p.-immunized with OVA plus Incomplete Freund’s Adjuvant (IFA) one month later.

**Results:**

Newborn piglets (n = 5/group) were gavaged with a combined soluble and particulate vaccine consisting of OVA (0.5-0.05 mg) plus 50 μg CpG and 0.5 mg OVA plus 50 μg CpG encapsulated within a polyphosphazene MP (0.5 mg) referred to as OVA/CpG + MP. Control piglets were gavaged with saline alone. Piglets were i.p. immunized with 10 mg OVA (or saline) in IFA at four weeks of age and then euthanized at eight weeks of age. We observed significantly higher titres of serum anti-OVA immunoglobulin (Ig) IgM, IgA, IgG, IgG1, IgG2 and IgG in piglets immunized with 0.05 mg OVA/CpG + MP relative to saline control animals. Thus, a single oral exposure at birth to a combined soluble and particulate OVA vaccine including adjuvants can circumvent induction of oral tolerance which impacts response to i.p. vaccination in later life. Further, piglets gavaged with 0.05 mg OVA/CpG + MP generated significant anti-OVA IgG and IgG1 titres in lung compared to saline control piglets but results were comparable to titres measured in parenteral control piglets. Peripheral blood mononuclear cells (PBMCs) *ex vivo*-stimulated with OVA showed markedly decreased production of IL-10 cytokine after 72 hours relative to animal-matched cells incubated with media alone. No production of IFN-γ was observed from any groups.

**Conclusion:**

Newborn piglets gavaged with low dose soluble and particulate OVA plus CpG ODN and polyphosphazene adjuvants produced antigen-specific antibodies in serum and lung after systemic re-exposure in later life. These data indicate circumvention of oral tolerance but not induction of oral immunity.

## Background

If exposure to an antigen by the oral route fails to promote an oral immune response, any subsequent re-exposure (even by a systemic route) results in suppression of immunity; this process is known as oral tolerance. Oral tolerance is a major suppressive immunological process designed to prevent local and peripheral overreaction to innocuous antigens [1,2]. Commensal bacteria are critically required for proper gut-associated lymphoid tissue (GALT) development and induction of oral tolerance [3,4]. Mucosal dendritic cells (DCs) play an active role in inducing oral tolerance through mechanisms which require retinoic acid, vitamin D, interleukin (IL)-10, Transforming growth factor (TGF)-β, and indoleamine-2,3,-dioxygenase [5-9]. In the mesenteric lymph nodes (MLNs), T regulatory (Treg) cells undergo differentiation and home back to the inductor site to induce and/or maintain antigen-specific oral tolerance [8]. Several physical barriers prevent antigen/pathogen contact with GALT and subsequent penetration of the gut wall making targeted induction of oral immunity a significant challenge [10,11]. The gut of the newborn piglet is uniquely designed to be semi-permeable or ‘leaky’ for a limited time to allow colostrum-derived cells, antibodies, and other macromolecules such as albumin, cytokines, antimicrobial peptides and many other bioactive products to be passively transferred to the piglets. These maternally-derived cells and macromolecules traverse the gut wall then enter into the vasculature where they play a variety of roles including passive immunity against disease [12-15]. ‘Gut closure’ occurs within a few days after birth in ruminants [16] and pigs [17], but it does not occur until after weaning (two weeks of age) in rats and mice [18-20]. In humans, a considerable amount of ‘gut-closure’ occurs both before birth and within a few days after birth but it may in fact take up to two years to reach the same level of impermeability that is observed in the adult gut [21,22]. Once across the gut wall, antigens may be able to interact with DCs within the sub-epithelial dome which can then present antigens to T cells within isolated lymphoid follicles and/or Peyer’s Patches to promote induction of oral immunity rather than being taken up by tolerogenic mucosal DCs which promote oral tolerance [23,24]. Despite the overwhelming propensity to respond to an oral antigen with tolerance, oral vaccines are highly sought because of their ease of administration. They are needle-free and therefore present reduced risk of transmitting infections and less need for qualified personnel to administer the vaccine. Moreover, an estimated 90% of all pathogens invade through mucosal surfaces, therefore mucosal immunity (induced by mucosal vaccines) offer the potential to control pathogens at their point of entry.

Previous work from our lab showed that rat pups and lambs orally vaccinated starting the day after birth with multiple doses of soluble ovalbumin (OVA; without adjuvants) responded with immunity to subsequent intraperitoneal (i.p.) immunization [25,26]. In contrast, we showed that newborn piglets orally immunized within six hours after birth with single bolus of soluble OVA then boosted through the i.p. route one month later showed significantly lower anti-OVA immunoglobulin (Ig) A titres and a strong trend towards lower anti-OVA IgM, IgG1, IgG2 and IgG titres relative to the i.p. control group indicating induction of oral tolerance [27]. These data showed agreement with Haverson et al [28] who demonstrated that newborn piglets orally vaccinated once with OVA induced classical oral tolerance following a systemic challenge by showing reduced specific systemic IgG responses. When we included unmethylated oligonucleotides containing CG oligodeoxynucleotides (CpG ODNs) and soluble polyphosphazene in the oral vaccine administered at birth, the response to i.p. immunization one month later was induction of immunity (i.e. increased serum anti-OVA IgA, IgM, IgG1, IgG2 and IgG titres relative to piglets immunized with OVA alone) [27]. Clearly the components of the oral vaccine administered at birth impacted the response to the booster immunization one month later.

Factors contributing to induction of oral tolerance include: the host’s immunological maturity at time of exposure, the timing and the frequency of exposure, and the nature of the antigen [29-32]. We established that a single bolus of soluble OVA with CpG ODN and polyphosphazene adjuvants administered the day after birth induced oral immunity in piglets [27]. Our next step will be to establish whether inclusion of the antigen in a particulate form promotes oral immunity and whether the response could be observed at distal mucosal sites. To test this hypothesis, conventionally reared neonatal piglets were gavaged within six hours after birth with 0.5 mg or 0.05 mg OVA plus CpG ODN in a soluble form as well as OVA plus CpG ODN encapsulated within a polyphosphazene microparticle (MP) [33-35]. Systemic and mucosal antibody titres and *ex vivo* cytokine production are assessed to determine antibody-mediated and cell-mediated immunity.

## Results

### Explanation of vaccination procedure

**Figure 1:**
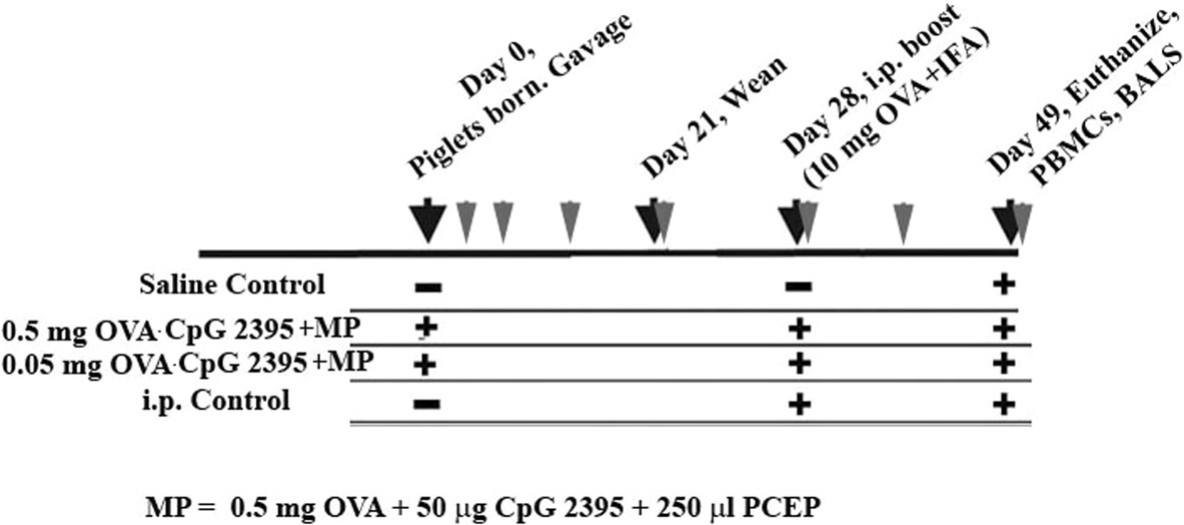
**Description and timeline of immunization protocol.** Piglets (n = 5/group) were gavaged with ovalbumin (OVA) (0.05 mg or 0.5 mg) + 50 μg unmethylated oligonucleotides containing CG oligodeoxynucleotides (CpG 2395) as a soluble vaccine as well as with 0.5 mg OVA + 50 μg CpG 2395 within a 0.5 mg PCEP polyphosphazene microparticles (MP). Gavages took place within six hours of birth. The negative control (saline) group and the i.p. control group were gavaged with saline. With the exception of the saline control group, the remaining groups were i.p. immunized at four weeks of age with 10 mg OVA in Incomplete Freunds’ Adjuvant (IFA). At eight weeks of age, piglets were euthanized and lung lavages were harvested. Blood was obtained on day three, day seven and then weekly (grey arrows). At time of death, blood was drawn for PBMC isolation.

Piglets were gavaged with a two-part vaccine consisting of soluble OVA (0.5 mg or 0.05 mg) with 50 μg soluble CpG 2395 (which together make up the soluble components of the vaccine; OVA/CpG) as well as a MP encapsulating 0.5 mg OVA + 50 μg CpG 2395 (which comprised the particulate part of the vaccine). These vaccines will be referred to as 0.5 mg OVA/CpG + MP and 0.05 mg OVA/CpG + MP. The experimental timeline is detailed in Figure 1. Piglets were gavaged at less than six hours of age when their gut would be semi-permeable with the idea that both the soluble OVA/CpG and the OVA and CpG ODN within the MP would cross the leaky gut wall. Polyphosphazene-based MPs are water-soluble and would then dissolve over time to release the encapsulated OVA and CpG ODN thus acting like a prime-boost [36,37]. Piglets were bled three days after birth, day seven after birth, and weekly thereafter (Figure 1, grey arrows specify bleed times). Piglets were i.p. immunized with 10 mg OVA (or saline) plus Incomplete Freund’s Adjuvant (IFA) at 28 days of age and all piglets were euthanized at 49 days of age. The i.p. control group received a saline gavage but piglets were boosted with the i.p. vaccination (OVA + IFA) to act as our primary i.p. vaccine control group.

### Newborn piglets vaccinated by oral gavage responded with significant serum anti-OVA IgM, IgA, and IgG1, IgG2 and IgG production after re-exposure by the i.p. route

**Figure 2:**
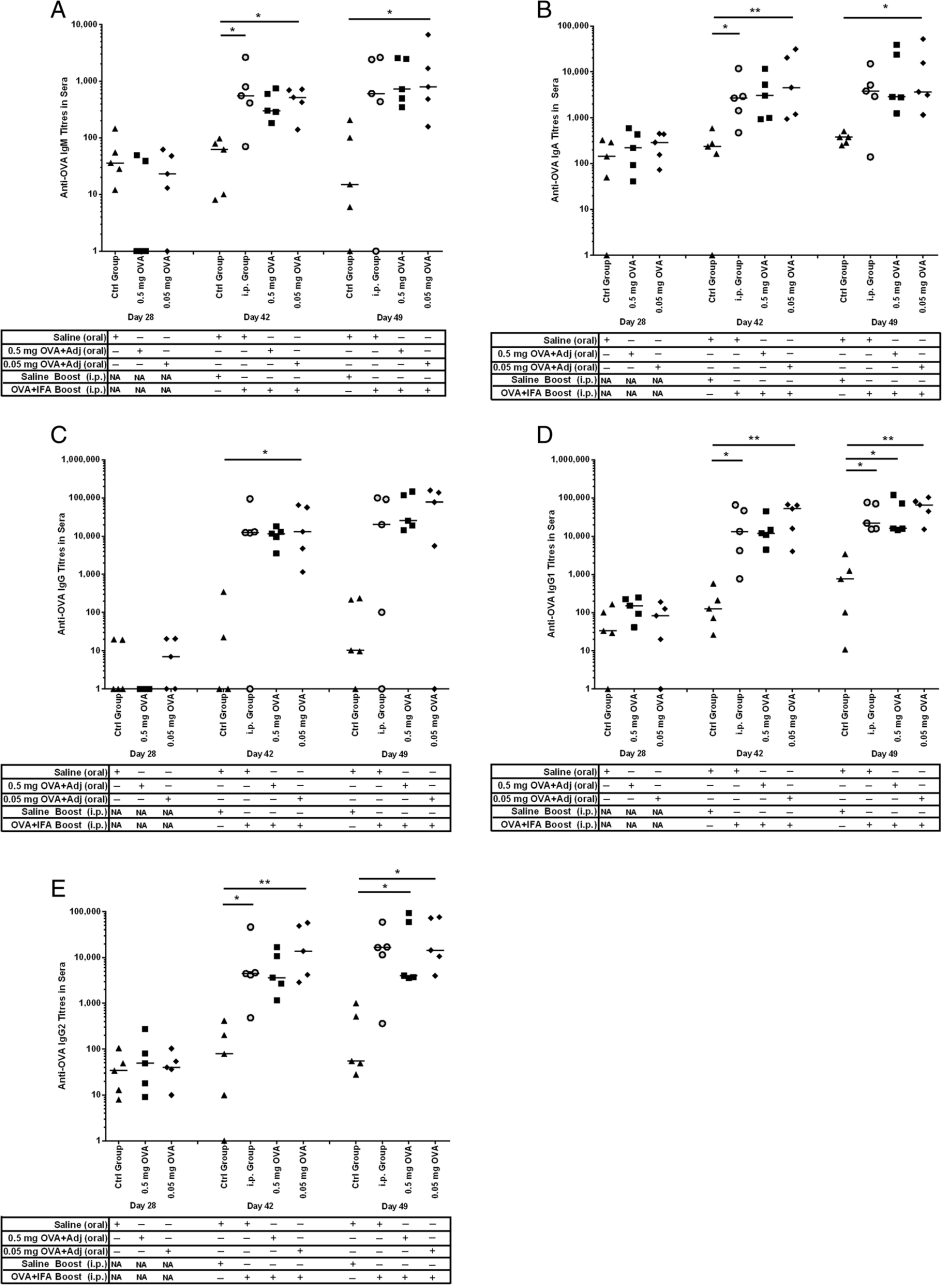
**OVA-specific antibody-mediated immune responses in serum from newborn piglets gavaged with OVA then i.p. immunized with OVA at four weeks of age.** Piglets (n = 5/group) were gavaged and i.p. immunized as described in Figure 1. Control newborn piglets were not gavaged or immunized with OVA. We measured serum anti-OVA IgM **(A)**, IgA **(B)**, IgG **(C)**, IgG1 **(D)** and IgG2 **(E)** production on day 28, day 42 and day 49 after birth. Each data point represents an individual animal and median values are indicated by horizontal lines. *p < 0.05., **p < 0.01.

The definition for oral tolerance is that oral exposure to antigen which is subsequently encountered via a systemic route triggers reduced immune responses (such as antibody production) relative to animals exposed to antigen systemically without prior oral exposure [38]. Previous work in our laboratory showed that animals orally immunized six hours after birth with 5 mg or 0.05 mg OVA responded to i.p. immunization at one month of age with oral tolerance, not oral immunity [27]. Therefore these groups (OVA alone without adjuvants) were not repeated here. In the current trial, when we assessed the serum antibody titres in the piglets prior to weaning (less than day 21) for all groups, we observed negligible anti-OVA antibodies of any isotype indicating that the sows did not pass any interfering OVA-specific passive immunity to the piglets (data not shown). Even one week after weaning (Figure 2, day 28), all isotypes of piglet serum anti-OVA antibodies titres were negligible suggesting that oral gavage at birth with 0.5 mg or 0.05 mg OVA/CpG + MP alone did not promote antibody-mediated immunity. On day 42, which was two weeks after the i.p. booster immunization, we observed a significant increase in serum anti-OVA IgM (Figure 2A; p < 0.05), IgA (Figure 2B; p < 0.05), IgG1 (Figure 2D; p < 0.05), and IgG2 (Figure 2E; p < 0.05) titres in the i.p. control group relative to the saline control group. The group gavaged with 0.05 mg OVA/CpG + MP showed significant induction of anti-OVA IgM (Figure 2A; p < 0.05), IgA (Figure 2B; p < 0.01), IgG (Figure 2C; p < 0.05), IgG1 (Figure 2D; p < 0.01), and IgG2 (Figure 2E; p < 0.01) relative to the saline control group suggesting that prior oral exposure to low dose OVA circumvented induction of oral tolerance. The animals gavaged with ten-fold higher dose of soluble OVA (0.5 mg OVA/CpG + MP) showed a strong trend towards increased anti-OVA antibody production relative to the saline control group but the data were not statistically significant.

On day 49, serum titres from animals within the i.p. control group showed a significant increase in anti-OVA IgG1 antibodies (Figure 2D; p < 0.05) relative to the saline control group. In contrast, the group gavaged with 0.05 mg OVA/CpG + MP showed significantly more anti-OVA IgM (Figure 2A; p < 0.05), IgA (Figure 2B; p < 0.05), IgG1 (Figure 2D; p < 0.01), and IgG2 (Figure 2E; p < 0.01) titres than what was observed in the saline control group. The group gavaged at birth with 0.5 mg OVA/CpG + MP showed significantly more anti-OVA IgG1 (Figure 2D; p < 0.05), and IgG2 (Figure 2E; p < 0.05) antibodies compared to the saline control group, but the other isotypes did not. Thus, unlike our previous data which showed that piglets orally vaccinated with soluble OVA alone induced oral tolerance [27], data from the current trial shows that piglets orally vaccinated with 0.5 mg or 0.05 mg OVA/CpG + MP showed significant induction of anti-OVA antibodies in serum indicating circumvention of oral tolerance.

### Newborn piglets vaccinated orally with low dose OVA/CpG + MP responded with significant anti-OVA IgG1 and IgG titres in lung lavage after re-exposure by the i.p. route

**Figure 3:**
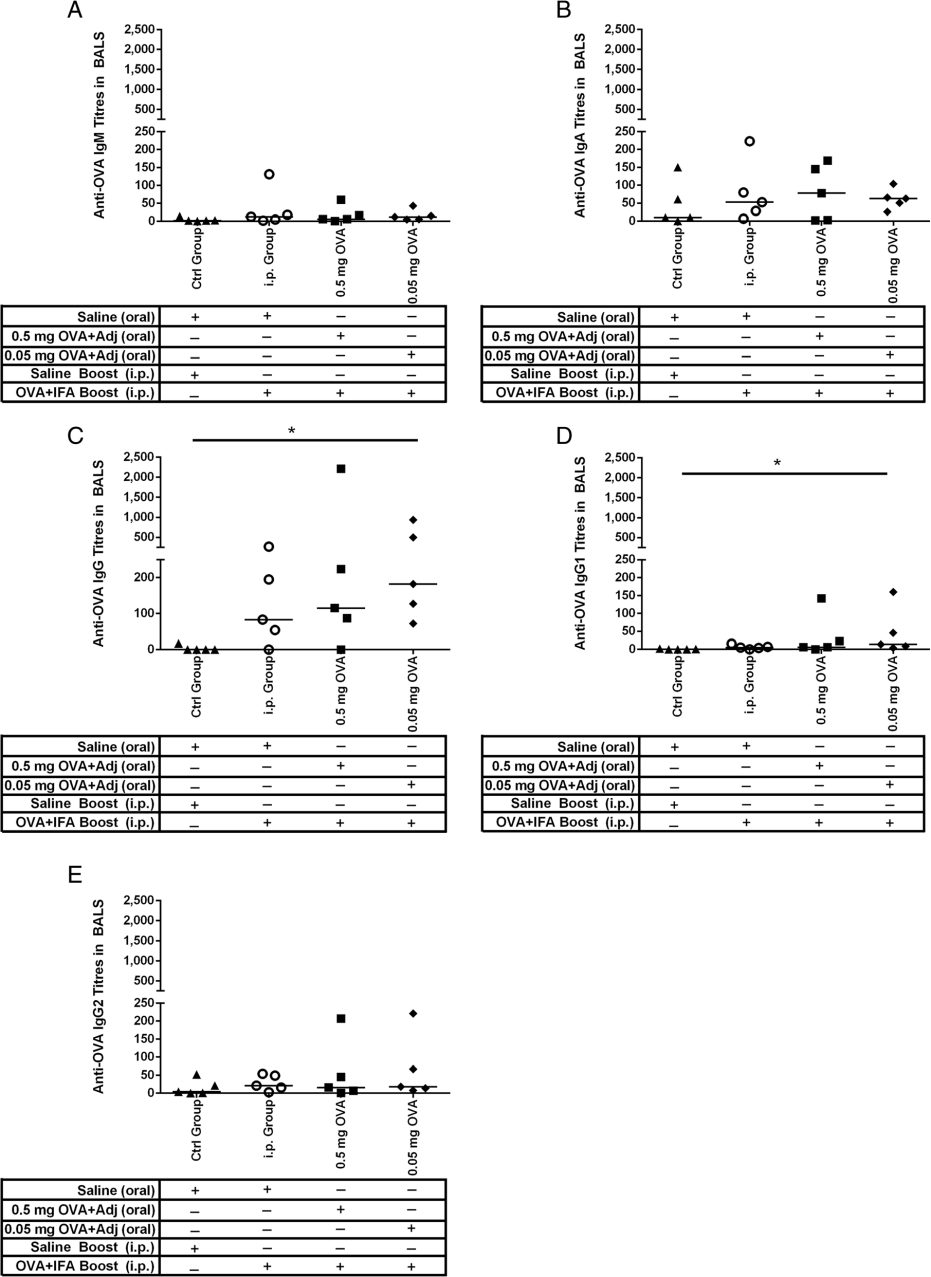
**OVA-specific antibody-mediated immune responses in lung washes from newborn piglets gavaged with OVA then i.p. immunized with OVA at four weeks of age.** Piglets (n = 5/group) were gavaged and i.p. immunized as described in Figure 1. Control newborn piglets were not gavaged or immunized with OVA. Lung lavages were collected four weeks post i.p. immunization and OVA-specific serum IgM **(A)**, IgA **(B)**, IgG **(C)**, IgG1 **(D)**, and IgG2 **(E)** titres were measured. ELISA titres are expressed as the reciprocal of the highest dilution resulting in a reading of two standard deviations above the negative control. Each data point represents an individual animal and median values are indicated by horizontal lines. *p < 0.05, **p < 0.01.

According to the ‘Common Mucosal Immune System’ theory, antigen-sensitized precursor B and T lymphocytes generated at one mucosal site (i.e. such as the gut) can be detected at anatomically remote and functionally distinct compartments (such as the respiratory mucosa) [39-45]. After 49 days, the pigs were euthanized and bronchoalveolar lavage was collected. Piglets gavaged with saline but injected with OVA and IFA by the i.p. route alone (i.e. the i.p. control group) failed to trigger significant anti-OVA IgM (Figure 3A), IgA (Figure 3B), IgG (Figure 3C), IgG1 (Figure 3D), or IgG (Figure 3E) titres relative to the saline control group. Newborn piglets gavaged with 0.05 mg OVA/CpG + MP had very low titres of anti-OVA IgM (Figure 3A), IgA (Figure 3B), and IgG2 (Figure 3E) but the titres for anti-OVA IgG (Figure 3C, p < 0.05) and IgG1 (Figure 3D; p < 0.05) were statistically higher than the saline control group. Collectively, these results suggest that oral exposure to OVA/CpG + MP triggered low level mucosal immunity at a distal site.

### Neonatal piglets gavaged with OVA/CpG + MP did not respond with induction of OVA-specific cell-mediated immunity

Finally, we sought to determine whether neonatal piglets orally gavaged with 0.5 mg or 0.05 mg OVA/CpG + MP developed cell-mediated immunity as measured by IFN-γ (Type 1 T-helper cell (Th1)) cytokines and IL-10 (Th2 cytokines). Peripheral blood mononuclear cells (PBMCs) were collected at eight weeks of age and they were restimulated *ex vivo* with OVA or media for 72 hours before the supernatants were collected and antibody titres were assessed. PBMCs did not show OVA-specific induction of IFN-γ for any group except in the presence of the mitogen Concanavalin A (data not shown). Interestingly, we observed a decrease in production of the anti-inflammatory cytokine IL-10 relative to unstimulated (media control) cells in all groups including the saline control groups. These data indicate that oral gavage of newborn piglets did not promote cell-mediated immunity as measured at 8 weeks of age.

## Discussion

Because the vast majority of infectious agents enter the body through mucosal routes, it is reasonable to assume that mucosal immunity which combats the infectious agent prior to colonization and penetration would be much more effective than systemic immunity. But it has proven very challenging to design effective oral subunit vaccine without the use of very strong mucosal adjuvants such as cholera toxin [46,47]. The majority of clinically approved oral vaccines for use in pigs are live attenuated viruses or bacteria (www.vetvac.org/index.php). One may speculate that in order to trigger an immune response instead of tolerance, the pathogen must traverse the gut wall and/or penetrate the epithelial cells lining the gut wall. If this is the case, it is understandable that subunit vaccines that lack strong adjuvants such as cholera toxin should fail to promote immunity [48]. However, such adjuvants cause significant side effects and are therefore not in clinical use. Due to the inherent risk of attenuated pathogen vaccines reverting to virulence, live attenuated vaccines against pathogens such as Porcine Reproductive and Respiratory Virus are not recommended for use in seronegative herds and are therefore a reactive vaccine instead of a proactive vaccine [49-51].

Previous work in our lab showed that oral administration of soluble OVA in rat pups or lambs starting immediately after birth was sufficient to promote oral immunity or at minimum prevented induction of oral tolerance [25,26]. In contrast, our research with piglets showed that soluble OVA administered immediately after birth triggered induction of oral tolerance [27]. However, if CpG ODN and polyphosphazene adjuvants were included in the soluble oral vaccine, there was instead evidence of induction of serum antibody-mediated immunity in response to systemic re-exposure in later life [27]. In the current study, we investigated whether oral immunization with a mixture of soluble and particulate OVA plus CpG ODN and polyphosphazene adjuvants could promote oral immunity. We determined that systemic anti-OVA antibody-mediated immune responses were induced in newborn piglets gavaged once on the day of birth with 0.5 mg or 0.05 mg OVA/CpG + MP and subsequently boosted with OVA and IFA by the i.p. route one month later. If oral tolerance to OVA had been induced instead, re-exposure to the antigen by i.p. injection should have resulted in a reduction of serum anti-OVA antibody titres as observed in [27]. Anti-OVA IgG and IgG1 titres in lung lavages were significantly induced in piglets gavaged at birth with 0.05 mg OVA/CpG + MP indicating induction of mucosal antibody-mediated immunity at a distal mucosal site. IgG has not traditionally been recognized as a major mucosal immunoglobulin, however there is growing evidence that oral vaccines can elevate local and systemic IgG titres [52,53] and that IgG antibodies may play a role in passive transfer of luminal antigens across the gut wall using FcRN [54]. Therefore, our data shows that vaccination of newborn piglets with a joint soluble and particulate subunit vaccine triggered systemic antibody-mediated immunity which contrasts with what is reported in older piglets orally vaccinated with soluble antigens or newborn piglets gavaged with OVA alone [27,52].

Results from our lab showed that oral vaccination of newborn lambs with OVA produced minimal cell-mediated immunity as established by IFN-γ expression and lymphocyte proliferation in *ex vivo* stimulated splenocytes [26]. Similarly, *ex vivo*-stimulated mLN cells from rat pups gavaged with OVA after birth failed to produce significantly higher IFN-γ titres relative to cells from pups gavaged after birth with saline [25]. In the current study, we gavaged piglets with soluble and particulate OVA plus polyphosphazene and CpG ODN, the latter of which is known to promote IFN-γ production [55]. Despite inclusion of CpG ODN in the oral piglet vaccine, IFN-γ production was negligible suggesting that oral immunization of newborns may not induce significant cell-mediated immunity.

Further studies must be undertaken to clarify the precise dose, vaccine formulation and timing of exposure required for induction of cellular immunity as well as including direct measurements of mucosal immunity. Experiments are underway to elucidate the kinetics of gut permeability and the impact this has on the mechanisms of antigen uptake and where antigen presentation to lymphocytes occurs (i.e. in the Peyer’s patches, isolated lymphoid follicles, or mesenteric lymph nodes). Should early life oral vaccination consistently circumvent induction of oral tolerance and/or promote oral immunity, it will have important implications for protecting against infectious diseases in the very young and it may reduce the number of carriers of disease-producing organisms within a herd.

## Conclusions

In the present study, we determined that low dose OVA/CpG + MP circumvented induction of oral tolerance with comparable serum anti-OVA antibodies relative to the animals gavaged with 0.05 mg OVA/CpG + MP relative to the i.p. control animals. There was a trend towards induction of mucosal immunity but the results were not statistically significant. These results are intriguing and should be studied further to establish whether it may be advisable to proactively orally vaccinate newborn piglets to prevent induction of oral tolerance to ensure that the they can respond appropriately to parenteral vaccines in later life.

## Methods

### Immunization procedure

This work was approved by the University of Saskatchewan’s Animal Research Ethics Board, and adhered to the Canadian Council on Animal Care guidelines for humane animal use. Pregnant Landrace-cross sows were housed at VIDO with *ad libitum* access to standard feed and water. Piglets were randomly assigned to treatment groups. We gavaged piglets with a single bolus of saline or 0.5 mg or 0.05 mg OVA (Sigma-Aldrich Canada Ltd, Oakville, ON, Canada) plus 50 μg CpG 2395 (soluble components; 5′- TCGTCGTTTTCGGCGCGCGCCG -3′ phosphorothioate oligodeoxy-nucleotide from Merial Limited (Lyon, France)), as well as the particulate component comprised of 0.5 mg OVA + 50 μg CpG 2395 + 500 μg poly [di(sodiumcarboxylatoethylphenoxy)-phosphazene] (PCEP) (synthesized by Idaho National Laboratory (Idaho Falls, ID, USA). (These doses were extrapolated from a successful oral vaccination with OVA in lambs and weight adjusted [26]). Microparticles were formulated as detailed in [34]. A total volume of 10 mL was administered via gavage using soft Nalgene Tubing with a monojet catheter tip (Fisher Scientific Ltd., Ottawa, ON) gently inserted into the back of the throat. Piglets were bleed on day three and day seven and then weekly until day 49 (Grey arrows, Figure 1). All blood samples were collected using Ethylenediaminetetraacetic acid (EDTA) Vacutainers (BD Biosciences-Canada, Mississauga, ON), centrifuged (4547 × *g*) and serum stored at −20°C until antibody titres were measured. At four weeks age, piglets were i.p.-injected with 10 mg OVA plus IFA (Sigma-Aldrich). To generate the parenteral control group, piglets received saline by oral gavage and they were i.p. immunization with OVA plus IFA at four weeks as indicated above. This route was used because it is considered relevant for stimulating the mucosal tissues [56,57]. On day 49, piglets were euthanized using 2 mL/10 lb body weight with Pentobarbital Sodium Injection (240 mg/mL; Euthanyl, Bimeda-MTC Animal Health Inc., Cambridge, ON). Lung lavages were obtained at the end of the trial (Eight weeks; Figure 1). The lung lavage was kept on ice until centrifuged at 400 × *g* at 4°C for 10 min, then cells were washed twice with cold PBS, counted, and suspended to a final concentration of 4 × 10^6^ cells/mL in 10% complete Roswell Park Memorial Institute (RPMI) medium (Gibco; Life Technologies, Burlington, Ontario), supplemented with 0.2 mM L-glutamine, 0.1 mM HEPES, 0.05 mg/mL gentamicin, 0.02 mM 2-mercaptoethanol (Sigma-Aldrich for all), and 10% heat inactivated horse serum (Gibco; Life Technologies). Cells were stored at -20°C until used for cytokine ELISAs. Endotoxin concentration in OVA was determined to be 8,000 U/ml using the Limulus Amebocyte Lysate enzymatic assay QCL-1000 (Lonza Group Ltd, Basel, Switzerland) according to the manufacturer’s instructions.

### Serum and lung lavages ELISA

To measure OVA-specific antibody titres, blood sera and lung lavages was obtained as indicated above and ELISAs were performed as previously described [33]. Immunolon II microtiter plates (Dynex Technology Inc., Chantilly, VA, USA) were coated overnight at 4°C with OVA at 500 μg/ml in carbonate coating buffer (15 mM Na_2_CO_3_, 35 mM NaHCO_3_, pH 9.6; Sigma-Aldrich) and 100 μL of antigen added to each well. Wells were washed six times with distilled H_2_O. Pig serum or lavage samples were diluted as appropriate in Tris-buffered saline plus 0.05% Tween (Sigma-Aldrich) then they were added to the wells at 100 μL/well and incubated for two hours at room temperature (RT). Wells were washed again with distilled H_2_O and mouse anti-porcine IgA (Ab Serotec, Raleigh, NC, #MCA 638, 1/300), mouse anti-porcine IgG1 (Ab Serotec, #MCA 635, 1/600), mouse anti-porcine IgG2 (Ab Serotec, #MCA 636, 1/300), or mouse anti-porcine IgM (Ab Serotec, #MCA 637, 1/100) were added to the wells in a 100 μL volume and incubated for one hour at RT. Wells were washed again with dH_2_O and goat anti-mouse IgG (H + L) alkaline phosphatase conjugated (KPL, Gaithersburg, MD, USA, #075-1806, 1/5000) was added to each well at 100 μL/well followed by incubation for one hour at room temperature (RT). One hundred microlitres of goat anti-porcine IgG alkaline phosphatase conjugated (KPL, Gaithersburg, MD, USA, #15-14-06, 1/5000) to each well was used for total IgG detection. Wells were washed six times in dH_2_O before di(Tris) p-nitrophenyl phosphate (PNPP; Sigma-Aldrich) was diluted 1 mg/mL in PNPP substrate buffer (1 mM of MgCl_2_, 200 mM of Tris-HCl, pH 9.8; Sigma-Aldrich) and 100 μL/well was added to the wells. The reaction was allowed to develop for 60 min before absorbance was read as optical density (OD) at 405 nm in a Microplate Reader (Bio-Rad Laboratories, CA, USA). Results were reported as titres which are the reciprocal of the highest dilution that gave a positive OD reading. A positive titre was defined as an OD reading that was at least two times greater than the values for a negative sample.

### PBMC Isolation and Bioplex Cytokine Assays

PBMCs were isolated following the protocol described by Buchanan et al [58]. Stimulation of PBMCs were performed in 96-well, round-bottom plates (Nunc, Naperville, Ill., USA) using AIM-V® medium supplemented with 10% fetal bovine serum (Invitrogen, Burlington, ON), 2 mM L -glutamine, 50 μM 2-mercaptoethanol and 10 μg/mL polymyxin B sulfate (Sigma-Aldrich for all) as described before [12]. For each treatment, 5x10^5^ cells were cultured for 72 h in triplicate wells with media alone or 20 μg/ml OVA in 200 μL total volume. Culture supernatants were harvested and stored at –20°C until assayed for IL-10 and IFN-γ using Bioplex Cytokine Assay.

Bioplex bead coupling was performed as per the manufacturer’s instructions. The reagents were as follows: Coating antibody: monoclonal anti-swine IL-10 (Invitrogen ASC0104), Detection antibody: monoclonal anti-swine IL-10 biotin (Invitrogen ASC9109), Standard: recombinant swine IL-10 (Invitrogen PSC0104) and Coating antibody: monoclonal anti-swine IFN-γ (Fisher Scientific Ltd, ENMP700), Detection antibody: monoclonal anti-swine IFN-γ (Fisher Scientific Ltd,ENPP700; biotinylated in-house), Standard: recombinant swine IFN-γ (Ceiba Geigy). The multiplex assay was carried out in a 96 well Grenier Bio-One Fluotrac 200 96 F black (VWR CanLab Mississauga, ON, #82050-754) which allows washing and retention of the Luminex beads. The porcine IFN-γ and porcine IL-10 protein standards were added to the wells at 50 μl per well at an initial concentration of 2000 pg/mL and 5000 pg/ml, respectively followed by two-fold dilutions to make a standard curve. The PBMC supernatants were prediluted 1:3 and added to the wells at 50 μL per well. The two beadsets conjugated with the IFN-γ and IL-10 antigens were vortexed for 30 s followed by sonication for another 30 s to ensure total bead dispersal. The bead density was adjusted to 1200 beads per μl in PBS-BN (1x PBS + 1% BSA (Sigma-Aldrich) + 0.05% sodium azide (Sigma-Aldrich), pH 7.4) and 1 μL of each beadset was added to 49 μl of the PBSA + 1% New Zealand Pig Serum (Sigma-Aldrich P3484) + 0.05% sodium azide (Sigma-Aldrich) which was then added to each well. The plate was sealed with plate sealer (Fisher Scientific Ltd, #12565491) and covered with foil lid. The plate was agitated at 800 rpm for one hour at room temperature. After one hour incubation with serum, the plate was washed using the Bio-Plex ProII Wash Station (Bio-Rad Laboratories; soak 20 s, wash with 150 μL PBST three times). A 50 μL cocktail consisting of porcine IFN-γ (Fisher Scientific Ltd, (Endogen); 1/300 (which was biotinylated in-house) and biotinylated porcine IL-10 (Invitrogen; 0.5 μg/mL) was added to each well. The plate was again sealed, covered and agitated at 800 rpm for 30 minutes at room temperature then washed again as indicated above. A 50 μL volume of Streptavidin R-Phycoerythrin (RPE) (diluted to 5 μg/mL; Cedarlane, Burlington, ON; # PJRS20) was added to each well. The plate was again sealed, covered and agitated at 800 rpm for 30 minutes at room temperature and washed as indicated above. A 100 μL volume of 1 × Tris-EDTA was added to each well and then the plate was vortexed for 5 minutes before reading on the Luminex100 xMAP™ instrument (Luminex, Toronto, ON) following the manufacturer’s instructions and as described in [59]. The instrument was set up to read beadsets in regions 43 and 28 for IFN-γ and IL-10, respectively. A minimum of 60 events per beadset were read and the median value obtained for each reaction event per beadset. For all samples the multiplex assay mean fluorescent intensity (MFI) data was corrected for subtracting the background levels.

### Statistical analysis

**Figure 4:**
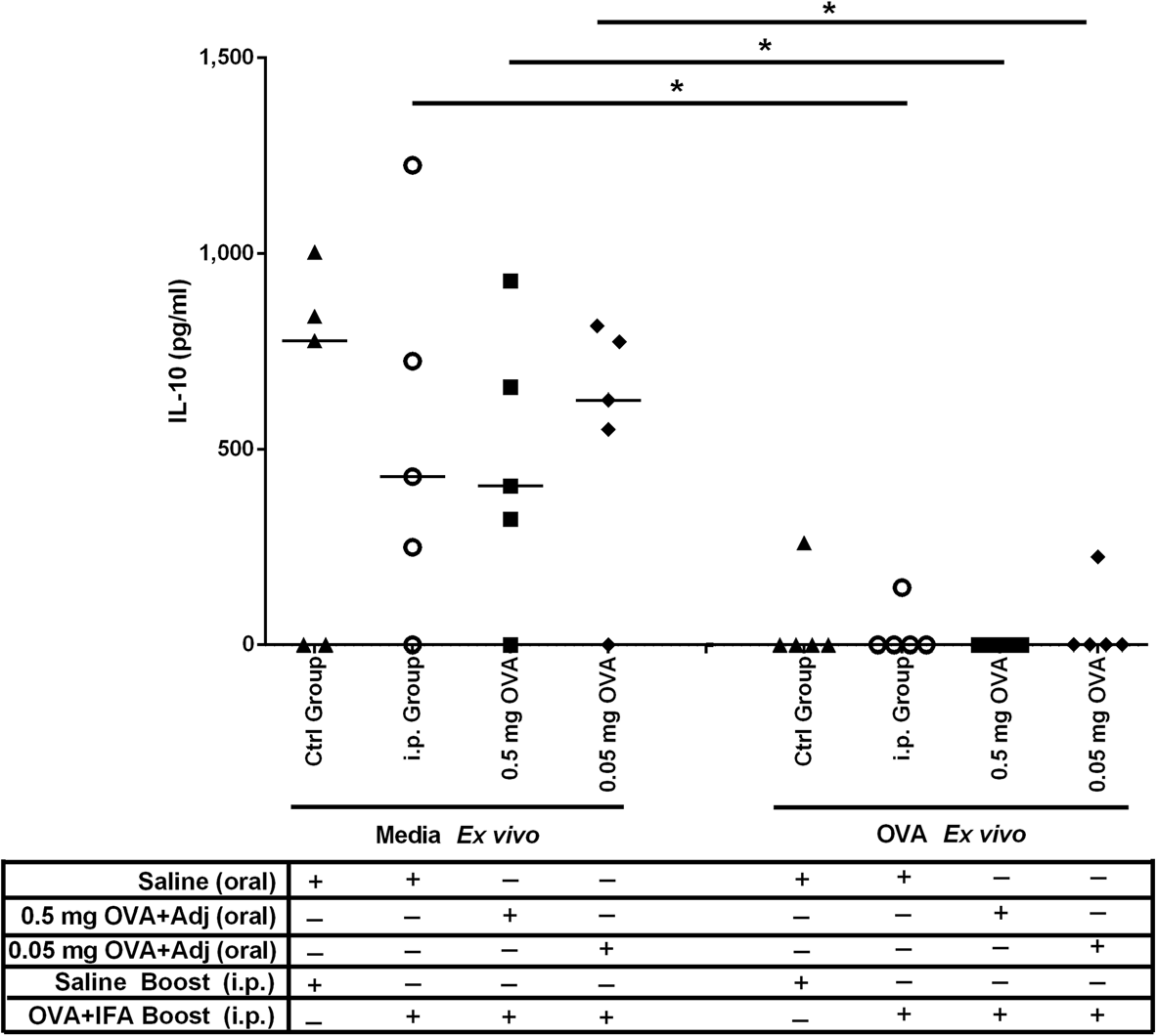
**OVA-specific cytokine production by PBMCs from piglets gavaged with OVA subunit vaccine then i.p. immunized with OVA/IFA at four weeks of age.** Piglets (n = 5/group) were gavaged and i.p. immunized as described in Figure 1. IL-10 production was measured from PBMCs obtained four weeks post i.p. immunization. PBMCs were re-stimulated with OVA or media ex vivo. After 72 hours, the supernatants were collected and measured by BioPlex assay. Each data point represents an individual animal and median values are indicated by horizontal lines.

The outcome data from this study were not normally distributed and therefore, differences among experimental groups were tested using Kruskal-Wallis analysis and medians were compared using Dunn’s test. In Figure 4, significance was determined using Mann-Whitney test. Differences were considered significant if p < 0.05. All statistical analyses and graphing were formed using GraphPad Prism 5 software (GraphPad Software, San Diego, CA).

## Abbreviations

BALs: Bronchoalveolar lavage
CMI: Cell-mediated immune response
DCs: Dendritic cells
PNPP: di(Tris) p-nitrophenyl phosphate
ELISA: Enzyme-linked immunosorbent assays
GALT: Gut-associated lymphoid tissues
h: hour
IL: Interleukin
IFA: Incomplete Freund’s Adjuvant
i.p: intraperitoneal
Ig: Immunoglobulin
IFLs: Isolated lymphoid follicles
MW: Molecular Weight
MFI: Mean fluorescent intensity
MLN: Mesenteric lymph node
MP: Microparticle
OVA: Ovalbumin
OD: Optical density
PBMC: Peripheral blood mononuclear cell
RPE: R-Phycoerythrin
RT: Room temperature
SIgA: Secretory IgA
StDev: Standard deviation
Th: T-helper cell
Treg: T regulatory cell
TBST: Tris-buffered saline with Tween 20
VIDO: Vaccine & Infectious Disease Organization
